# Adolescent Vitamin D Supplementation Reverses Neuroplasticity and Motivational Deficits Induced by Developmental Alcohol Exposure and Early‐Life Stress

**DOI:** 10.1002/jdn.70099

**Published:** 2026-01-30

**Authors:** Anelise Pereira Alves, Claudia Daniele Bianco, Clarice Mariano Fernandes, Ian Carlos Hübner, Patricia S. Brocardo

**Affiliations:** ^1^ Neuroscience Graduate Program, Center of Biological Sciences Federal University of Santa Catarina Florianopolis Brazil; ^2^ Morphological Sciences Department Federal University of Santa Catarina Florianopolis Brazil

**Keywords:** adolescent, early‐life stress, foetal alcohol spectrum disorder, hippocampal neuroplasticity, vitamin D

## Abstract

Early postnatal ethanol exposure (PNEE) and early‐life stress (ELS) are major contributors to persistent deficits in cognition, motivation and emotional regulation. These insults disrupt hippocampal plasticity and increase vulnerability to psychiatric disorders. Vitamin D (VitD), a neuroactive steroid, has emerged as a potential modulator of neurodevelopment and plasticity. We investigated whether adolescent VitD supplementation could mitigate behavioural and neuroplastic impairments resulting from early exposure to ethanol and maternal separation. On postnatal day (PND) 2, 64 Wistar rat pups (male and female) were randomized into eight experimental groups (*n* = 8 animals per group, with sex balanced across groups): (1) control, (2) VitD, (3) ethanol (EtOH), (4) EtOH + VitD, (5) maternal separation (MS), (6) MS + VitD, (7) EtOH + MS, and (8) EtOH + MS + VitD. EtOH groups received 5 g/kg i.p. ethanol on alternate days from PND 4–10. MS groups were separated from the dam for 3 h/day from PND 2–14. From PND 22–37, VitD groups received 1000 IU/kg/day of cholecalciferol. Behavioural assessments included palatable food intake and reward omission–based task. Brains were processed for doublecortin (DCX) immunohistochemistry and Golgi‐Cox analysis. EtOH + MS animals displayed increased latency to eat, reduced food consumption and persistent feeder‐directed responding following reward omission. VitD treatment reversed these effects, improving motivational performance and reducing reward omission–induced responding. VitD also restored hippocampal neurogenesis and normalized dendritic complexity and length. Vitamin D supplementation during adolescence mitigates behavioural and neuroplasticity deficits induced by PNEE (corresponding to the third trimester of human brain development) and ELS. These findings support VitD as a promising therapeutic strategy for neurodevelopmental disorders such as foetal alcohol spectrum disorder (FASD).

## Graphical abstract

Adolescent vitamin D supplementation reverses behavioural and neuroplastic impairments induced by early‐life alcohol exposure and maternal separation in rats. Postnatal ethanol exposure (PND 4–10) and maternal separation stress (PND 2–14) led to motivational deficits and altered hippocampal plasticity. Vitamin D administration during adolescence (PND 22–37) restored motivation, increased DCX + cell count in the dentate gyrus, and normalized dendritic complexity.

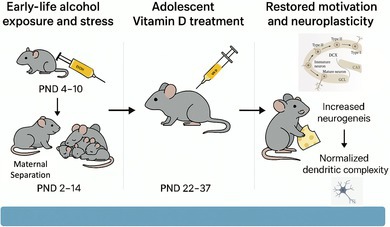

## 1 Introduction

Foetal alcohol spectrum disorder (FASD) is a neurodevelopmental condition caused by prenatal alcohol exposure (PAE) that leads to persistent cognitive, emotional and behavioural impairments, along with structural alterations in the brain (Popova et al. 2023). PAE disrupts critical neurodevelopmental processes, including epigenetic regulation, neuroendocrine function and synaptic plasticity, increasing susceptibility to psychiatric disorders (Ciafrè et al. 2020; Dong et al. 2018; Mattson et al. 2019).

These neurodevelopmental disruptions are frequently compounded by adverse postnatal environments. Individuals with FASD are disproportionately exposed to early‐life stress (ELS), including chronic neglect, caregiver instability, foster placements and socioeconomic adversity, which further exacerbate cognitive and emotional dysfunction (Burd et al. 2003; Lebel et al. 2019; Petrenko et al. 2017; Streissguth 1991; Streissguth et al. 2004). Extensive clinical and preclinical evidence demonstrates that ELS has a profound impact on neurodevelopment, particularly when superimposed on the vulnerabilities induced by PAE. This interaction leads to compounded disruptions in neural plasticity, stress responsivity and emotional regulation, resulting in long‐lasting behavioural impairments (Alberry et al. 2021; Raineki et al. 2014).

Importantly, ELS encompasses a heterogeneous set of experiences that vary in timing, duration and nature. In preclinical research, maternal separation is widely used as a controlled model of very early postnatal caregiver disruption, rather than a direct analogue of later childhood or adolescent adversity. Within models of FASD, early postnatal maternal separation has been shown to exacerbate neurodevelopmental and behavioural alterations induced by developmental alcohol exposure, highlighting the interaction between early biological vulnerability and subsequent environmental stressors (Alberry et al. 2021). Clinical research consistently shows that individuals with FASD who experience ELS exhibit markedly higher rates of psychiatric disorders, academic failure, substance abuse, involvement with the criminal justice system and persistent difficulties in achieving stable employment and independent living (Petrenko et al. 2017). Together, these findings support a ‘dual‐hit’ framework in which early alcohol exposure increases susceptibility to later environmental challenges, underscoring the need for interventions targeting this high‐risk population.

Adolescence represents a particularly sensitive period of neurodevelopment, marked by extensive synaptic pruning, dendritic remodelling and maturation of corticolimbic networks, especially in the hippocampus and prefrontal cortex—regions critical for emotional regulation, motivation and executive function (Spear 2000; Andersen 2003). This stage confers both heightened vulnerabilities to earlier developmental insults and a unique opportunity for therapeutic interventions that may harness neuroplasticity to improve outcomes. Importantly, many secondary disabilities associated with FASD—including academic failure, legal issues, substance misuse and challenges in social integration—tend to emerge or intensify during adolescence (Mattson et al. 2019; Petrenko et al. 2017; Streissguth et al. 2004).

In this context, increasing attention has turned to vitamin D, a neuroactive secosteroid traditionally recognized for its role in calcium homeostasis but now widely recognized as a critical modulator of brain development, synaptic plasticity and neuroimmune regulation (Eyles et al. 2013; Maddock et al. 2017). Vitamin D regulates key neurobiological processes, including neurogenesis, dendritic growth, neurotransmission and inflammatory modulation (Rodgers et al. 2023). Notably, experimental evidence indicates that ethanol exposure disrupts vitamin D metabolism and signalling by promoting proteasome‐mediated degradation of vitamin D receptors, particularly during sensitive periods of hippocampal development (Feltes et al. 2014). This disruption may contribute to the cognitive and emotional dysfunctions observed in FASD and may render the developing brain more vulnerable to subsequent stressors.

Given the central role of the hippocampus in motivation, emotional regulation and stress responsivity—and its heightened vulnerability to both PAE and ELS (Groves et al. 2014; Gáll and Székely 2021)—the present study investigates whether vitamin D supplementation during adolescence can mitigate neurobehavioural and neuroplasticity deficits induced by early alcohol exposure and maternal separation. Specifically, we assessed motivational behaviour, hippocampal neurogenesis and dendritic arborization in adolescent rats exposed to this dual‐hit developmental model. To our knowledge, this is the first study to evaluate vitamin D as a potential therapeutic strategy targeting the combined effects of developmental alcohol exposure and early postnatal caregiver disruption.

## 2 Methods

### 2.1 Animals

Male and female Wistar rats were obtained from the institutional breeding facility and housed under controlled conditions (12 h light/dark cycle, temperature 22°C ± 2°C), with food and water available ad libitum. Litters were culled to standardize litter size when necessary, and only one pup per litter was assigned to each experimental group.

A total of 64 pups were distributed across eight experimental groups (*n* = 8 animals per group). Sex was balanced across groups but was not included as an independent factor in the statistical analyses, as the study was not powered to detect sex‐specific effects. Accordingly, the individual animal was considered the experimental unit for all analyses.

Following weaning on PND 21, animals were housed in same‐sex groups of up to five per cage (opaque polypropylene cages, 41 × 34 × 16 cm) under standard laboratory conditions (temperature 20°C ± 2°C; 12‐h light/dark cycle, lights on at 07:00), with ad libitum access to food (Nuvilab CR1, Nuvital) and filtered water. All experimental procedures were conducted during the light phase between 09:00 and 17:00. The study was approved by the UFSC Committee on Animal Use (CEUA; protocol number 6980201116) and carried out in accordance with institutional, national and international ethical guidelines for animal research.

### 2.2 Experimental Protocol

**FIGURE 1:**
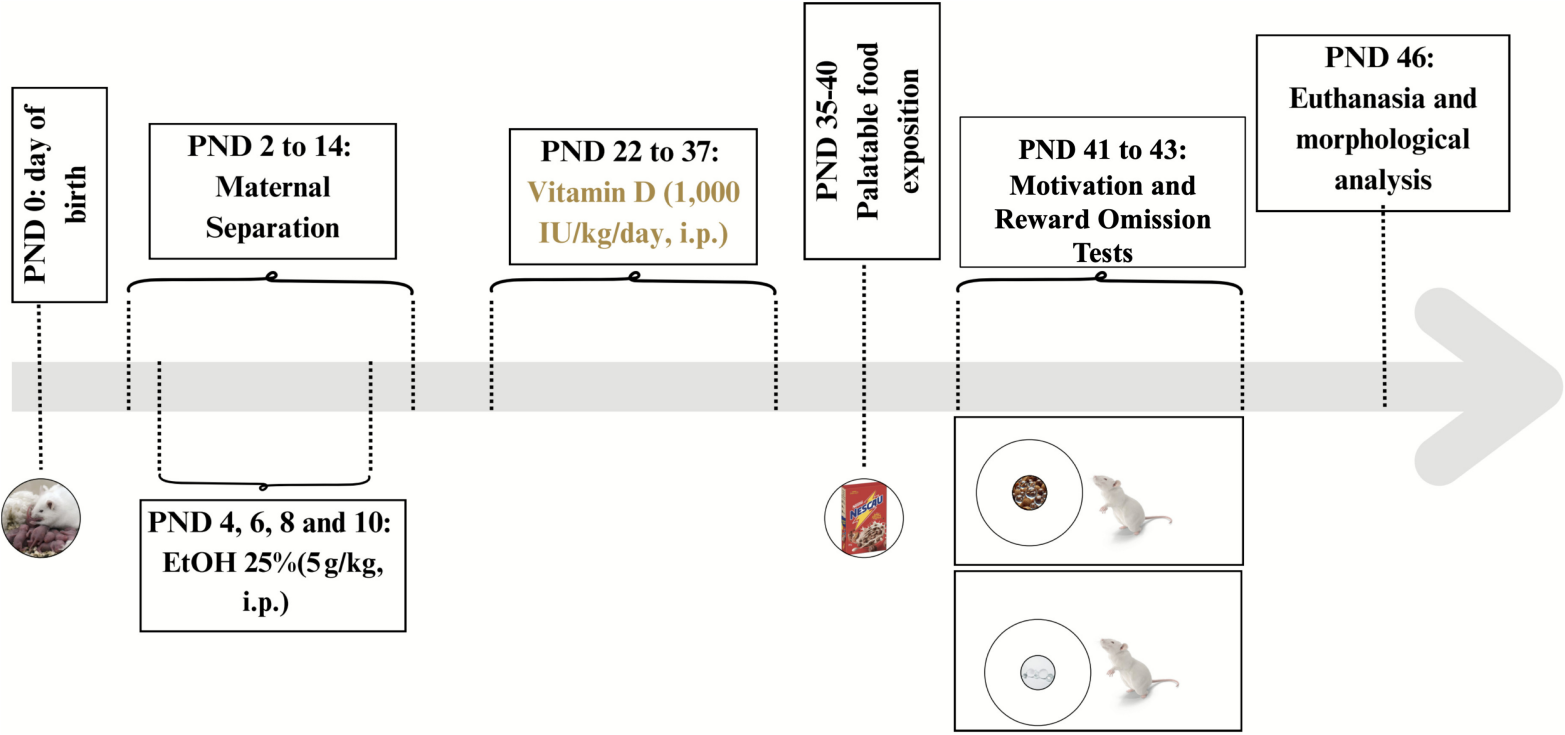
Experimental protocol timeline. The timeline illustrates the different interventions applied to the experimental groups throughout the study. From postnatal day (PND) 2 to PND 14, pups in the maternal separation (MS) group were separated from the dam for 3 h daily. Neonatal ethanol (EtOH) exposure was administered via intraperitoneal injections on PND 4, 6, 8 and 10. From PND 22 to PND 37 (adolescence period), animals received daily intraperitoneal injections of vitamin D or vehicle. Behavioural assessments for motivational behaviour and reward omission–induced responding were conducted between PND 41 and PND 44. On PND 46, animals were euthanized, and brains were collected for histological and morphological analyses.

On PND 2, pups from each litter were sexed, and one male and one female from each litter were randomly assigned to different experimental groups. The experimental design included eight groups (*n* = 8 animals per group), with sex balanced across groups but not treated as an independent analytical factor, as follows: (1) control, (2) vitamin D (VitD), (3) ethanol (EtOH), (4) EtOH + VitD, (5) maternal separation (MS), (6) MS + VitD, (7) EtOH + MS, and (8) EtOH + MS + VitD. Thus, the experimental design followed a 2 × 2 × 2 factorial structure, allowing the evaluation of main effects and interactions between early alcohol exposure, early postnatal stress and adolescent intervention. A schematic representation of the experimental timeline and interventions is shown in Figure 1.

### 2.3 Early Alcohol Exposure Protocol

From PND 4 to PND 10—corresponding to the third trimester of human gestation—pups in the EtOH groups received intraperitoneal (i.p.) injections of ethanol (5 g/kg, 25% solution in saline) on alternate days to simulate binge drinking behaviour observed in humans. Control group pups received equivalent volumes of saline solution via i.p. injection. This protocol results in an average blood alcohol concentration (BAC) of approximately 239 mg/dL, indicating a high level of alcohol exposure (Filgueiras et al. 2010). BAC was not directly measured in the present cohort and is acknowledged as a methodological limitation.

### 2.4 Maternal Separation Protocol

Entire litters were randomly selected and subjected to the MS protocol (MS) from PND 2 to PND 14 for 3 h/day (09:00–12:00), while control group litters were not separated from the mother (nMS, *n* = 40). The pups in the MS group were carefully removed from their cage and placed in a clean cage in a separate heated room (±25°C) to prevent communication between the pups and the mother. After 3 h, the pups were returned to their home cage with the mother (Adapted from Swart et al. 2019). This procedure was employed as a model of very early postnatal caregiver disruption and not as a direct analogue of later childhood or adolescent stress.

### 2.5 Vitamin D Treatment

Between PND 22 and PND 37, approximately 6 to 11 years of human age (Sengupta 2013), animals received daily i.p. injections of cholecalciferol (VitD, Webber Naturals Pharmaceuticals) at a dose of 1000 international units (IU)/kg/day (Liang et al. 2018). Injections were administered each day between 9:00 and 11:00 AM. to minimize circadian variability. Control groups received the vehicle (100% mineral oil; Purulim—Lifar). All experimental groups received daily intraperitoneal injections during this period (vitamin D or vehicle), thereby controlling for potential effects of repeated handling and injection‐related stress.

### 2.6 Behavioural Analyses

#### 2.6.1 Motivational Analyses

Motivational behaviour was assessed using a task designed to evaluate the animals' goal‐directed responses toward a natural hedonic stimulus (Becker and Bernecker 2023). The test was conducted in a transparent plexiglass arena (40 × 40 × 40 cm) containing a central feeder. Animals were not food‐deprived prior to testing to ensure that behavioural responses reflected hedonic motivation rather than caloric need.

The feeder contained both palatable food (sweetened cereal) and small plastic beads uniformly mixed within the feeder tray. This setup required the animals to actively search for and retrieve the palatable food from among the beads, increasing the demand for motivated goal‐directed behaviour while maintaining the ecological validity of the task. While this specific configuration was developed for the current study, it conceptually aligns with validated paradigms assessing motivation and reward sensitivity in rodents, particularly those that incorporate effort‐related components (Scheggi et al. 2018).

This behavioural protocol was adapted from a previously developed approach by Barbosa (2017), in which nonfood‐deprived rats were provided with palatable food (approximately 3 g of Nescau Ball Cereal per animal/day) in their home cages during the week preceding the test. Testing occurred between postnatal days (PND) 41–43, with *n* = 8 animals per group. The task consisted of two experimental phases separated by a 24‐h interval: (i) **Adaptation**—three 5‐min sessions of free exploration in the test apparatus without food; and (ii) **Test**—three 5‐min sessions in the same arena with the feeder containing 10 partially buried units of Nescau Ball Cereal among glass beads. The following parameters were recorded: (i) latency to initiate eating (time from placement in the arena to the first consumption of a food item), (ii) total number of palatable food units consumed and (iii) number of approaches to the feeder, indicating sustained engagement in the task.

Each animal was allowed to explore the arena for 5 min. The following parameters were recorded: (i) latency to initiate eating (time from placement in the arena to the first consumption of a food item), (ii) total number of palatable food units consumed and (iii) number of approaches to the feeder, indicating sustained engagement in the task.

Motivational behaviour was evaluated using a task designed to measure goal‐directed responses toward a natural hedonic stimulus, based on a previously developed protocol (Barbosa 2017; see also Becker and Bernecker 2023). The test was conducted in a transparent plexiglass arena (40 × 40 × 40 cm) equipped with a central feeder. Importantly, animals were not food‐deprived prior to testing to ensure that behavioural responses reflected hedonic motivation rather than caloric need. During the week preceding the test (postnatal days [PND] 41–43), animals received a small daily amount (~3 g) of palatable food (Nescau Ball Cereal) in their home cages to promote familiarity with the reward. On the test day, each animal was allowed to explore the arena during three 5‐min sessions. The feeder contained 10 units of sweetened cereal partially buried among inert objects (glass or plastic beads), requiring the animal to actively search for and retrieve the food. This setup increased the effort needed to obtain the reward while maintaining ecological validity and aligning with validated paradigms assessing motivation and reward sensitivity in rodents (Scheggi et al. 2018).

The task was composed of two phases separated by a 24‐h interval: (i) Adaptation phase—three 5‐min sessions in the arena without any food present, allowing habituation to the environment; and (ii) Test phase—three 5‐min sessions with the baited feeder. The following parameters were recorded: Latency to initiate eating (time from placement in the arena to the first consumption of a food item); total number of palatable food units consumed; number of feeder approaches, indicating sustained engagement in the task.

#### 2.6.2 Reward Omission Task

To evaluate behavioural responses following reward omission, animals were re‐exposed to the same arena 24 h later for a single 5‐min session in which the feeder contained only plastic beads, thereby omitting the previously available palatable reward. Behavioural outcomes are described as reward omission–induced responding, operationalized as persistent feeder‐directed behaviour under nonreinforcement, without inferring a specific emotional state. The following parameters were analysed: latency to explore the feeder, number of entries into the feeder area and total time spent exploring the feeder.

### 2.7 Evaluation of Hippocampal Neuroplasticity

#### 2.7.1 Brain Tissue Processing

At postnatal day (PND) 46, animals were deeply anaesthetised with an intraperitoneal injection of ketamine (100 mg/kg) and xylazine (10 mg/kg) and transcardially perfused with 0.9% saline solution followed by 4% paraformaldehyde (PFA) in phosphate‐buffered saline (PBS) for tissue fixation. Following perfusion, brains were carefully extracted from the skull and postfixed in 4% PFA at 4°C for 12 h. Subsequently, brains were cryoprotected by immersion in a 30% sucrose solution in PBS until they were fully saturated (indicated by sinking).

Coronal sections containing the hippocampus were obtained using a vibratome (Vibratome Series 1000, St. Louis, MO, USA) at a thickness of 30 μm. Sections were collected in a 1:6 series systematically spanning the entire rostrocaudal axis of the hippocampus and stored in PBS containing 0.5% sodium azide at 4°C until further processing for immunohistochemistry.

#### 2.7.2 Immunostaining for Neuronal Differentiation

Coronal sections containing the hippocampus were immunostained for doublecortin (DCX), a marker of immature neurons (Ayanlaja et al. 2017), in a subset of animals (*n* = 5–6 per experimental group). Free‐floating sections were incubated in 3% hydrogen peroxide with 10% methanol in TBS for 15 min to block endogenous peroxidase, followed by 1 h in 5% horse serum to prevent nonspecific binding.

Sections were then incubated with a goat polyclonal anti‐DCX antibody (1:400; Santa Cruz Biotechnology) at 4°C for 48 h. After rinsing, they were incubated with a biotinylated horse antigoat secondary antibody (1:200; Vector Laboratories) for 2 h. Immunoreactivity was visualized using an avidin‐biotin–peroxidase complex (ABC Kit, Vector) and developed with DAB (Sigma‐Aldrich) for approximately 3 min. Sections were mounted on gelatin‐coated slides, dehydrated, cleared in xylene, and coverslipped with Entellan (Merck, USA) for subsequent analysis.

Quantification of DCX+ cells was performed in the subgranular zone (SGZ) of the dentate gyrus (DG) as a measure of adult hippocampal neurogenesis. High‐resolution images were acquired using an Olympus BX53 microscope at 10× magnification for global quantification and 40× for morphological confirmation. DCX+ cells were manually counted bilaterally along the entire SGZ across a systematic 1:6 series of coronal sections spanning the full rostrocaudal axis of the DG. Counts were expressed as the average number of DCX+ cells per section. All analyses were performed blind to the experimental groups.

#### 2.7.3 Golgi Staining for Dendritic Arborization

A subset of animals (*n* = 3 animals per experimental group) was processed for Golgi‐Cox staining to evaluate dendritic arborization in the DG of the hippocampus, following the protocol adapted from Kannangara et al. (2015). Brains were immersed in Golgi‐Cox solution for 15 days and then cryoprotected in 30% sucrose.

Coronal sections (200 μm thick) were obtained using a vibratome and incubated in a humid chamber for 48 h. Sections were then rinsed twice in double‐distilled water (2 min each), followed by incubation in 18% ammonium hydroxide for 10 min in the dark at room temperature. After six washes in double‐distilled water (5 min each), the stain was fixed in 1% sodium thiosulfate for 10 min in the dark, followed by six additional washes.

Finally, sections were dehydrated in ascending ethanol series, cleared in xylene and cover slipped with Entellan (Merck) for microscopic analysis.

#### 2.7.4 Dendritic Morphology Analysis

Dendritic arborization of granule cells in the dentate gyrus (DG) was quantified using Sholl analysis (ImageJ software's Simple Neurite Tracer plugin). Neurons with minimal overlap and well‐defined dendritic branches were chosen for analysis. Imaging was performed using an Inverted Microscope IX43 (Olympus) at 40× magnification. Dendrites were manually traced to create a 3D representation of each neuron, allowing for the quantification of branch lengths and the number of intersections with concentric circles. Concentric circles at 10‐μm intervals were centred on the soma to analyse parameters such as dendritic intersections per radius, total number of intersections and dendritic length (maximum distance from the soma to dendrite tips). For each experimental group (*n* = 3 animals per group), five neurons in the DG region per animal were randomly selected. Neuron‐level measurements were averaged within each animal, and the resulting animal means were used for statistical analyses, thereby avoiding pseudoreplication.

### 2.8 Statistical Analysis

The results were analysed using Statistica 7.0 software (StatSoft Inc., Tulsa, OK, USA). For all outcome measures, data were analysed using a three‐way analysis of variance (ANOVA) with the following fixed factors: condition (EtOH vs. saline), stress (MS vs. nMS) and treatment (VitD vs. vehicle). The individual animal was defined as the experimental unit in all analyses. Behavioural data were analysed with *n* = 8 animals per experimental group. For immunohistochemical analyses of doublecortin (DCX), a subset of animals was used (*n* = 5–6 animals per group). For Golgi‐Cox–based dendritic analyses, *n* = 3 animals per group were included, with neuron‐level measures averaged per animal prior to statistical testing to avoid pseudoreplication. Sex was balanced across groups but was not included as an analytical factor. When significant main effects or interactions were detected, post hoc comparisons were performed using Duncan's multiple range test. A *p*‐value of less than 0.05 was considered statistically significant.

## 3 Results

### 3.1 Effects of Vitamin D on Palatable Food Intake in Nonfood‐Deprived Rats

**FIGURE 2:**
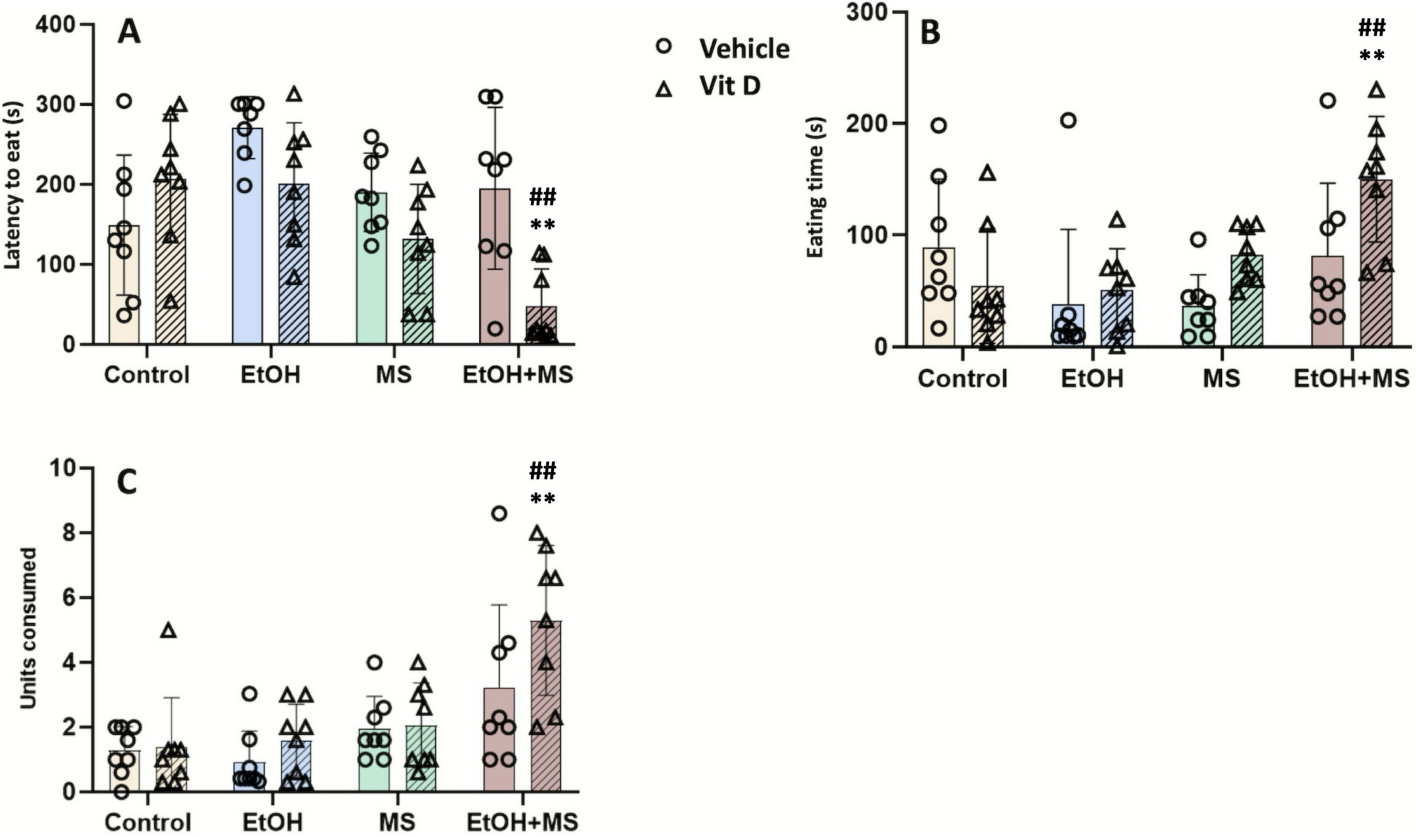
Motivational responses across experimental groups. (A) Latency to eat; (B) eating time; (C) number of palatable food units consumed. Data are expressed as mean ± SEM (*n* = 8 animals per group). **p < 0.01 compared to the control group (vehicle‐treated, saline, nonstressed); #*p* < 0.05, ##*p* < 0.01 compared to the corresponding vehicle‐treated group within the same condition. Abbreviations: EtOH + MS, ethanol plus maternal separation; MS, maternal separation; VitD, vitamin D.

The effects of vitamin D supplementation on motivated behaviour were assessed using a palatable food intake task in nonfood‐deprived rats (Figure 2). All behavioural analyses were conducted using the individual animal as the experimental unit (*n* = 8 animals per group). Multifactorial ANOVA revealed significant main effects of stress (MS vs. nMS) [*F*(1.55) = 13.90, *p* < 0.001] and treatment (vitamin D vs. vehicle) [*F*(1.55) = 9.44, *p* = 0.003]. Additionally, significant interactions were observed between condition (EtOH vs. saline) and stress [*F*(1.55) = 7.57, *p* = 0.008], condition and treatment [*F*(1.55) = 9.44, *p* = 0.003] and stress and treatment [*F*(1.55) = 7.56, *p* = 0.008]. Post hoc analyses indicated that rats exposed to EtOH alone (EtOH + nMS + vehicle) exhibited the longest latency to initiate eating, significantly higher than the control group (*p* < 0.01). In contrast, rats subjected to both EtOH and MS and treated with vitamin D (EtOH + MS + VitD) showed the shortest latency to eat (Figure 2A), with significant differences compared to both the nontreated EtOH + MS group (*p* < 0.001) and the control group (*p* < 0.001), indicating improved performance in this task following vitamin D treatment.

For the eating time parameter (Figure 2B), ANOVA revealed significant main effects of stress [*F*(1.55) = 5.12, *p* = 0.03] and significant interactions between condition and stress [*F*(1.55) = 10.39, *p* = 0.002] and between stress and treatment [*F*(1.55) = 6.91, *p* = 0.01]. Post hoc comparisons showed that the EtOH + MS + VitD group spent significantly more time eating palatable food compared to both its nontreated counterpart (*p* < 0.01) and the control group (*p* < 0.001), suggesting increased engagement with the food stimulus following vitamin D administration.

Analysis of the total number of food units consumed (Figure 2C) revealed significant main effects of condition [*F*(1.55) = 7.21, *p* = 0.01] and stress [*F*(1.55) = 20.48, *p* < 0.001], as well as a significant interaction between condition and stress [*F*(1.55) = 8.12, *p* = 0.006]. Rats exposed to EtOH and MS but treated with vitamin D (EtOH + MS + VitD) consumed significantly more units of palatable food compared to both the EtOH + MS + vehicle group (*p* < 0.01) and the control group (*p* < 0.001).

### 3.2 Effects of Vitamin D on Reward Omission–Induced Responding

**FIGURE 3:**
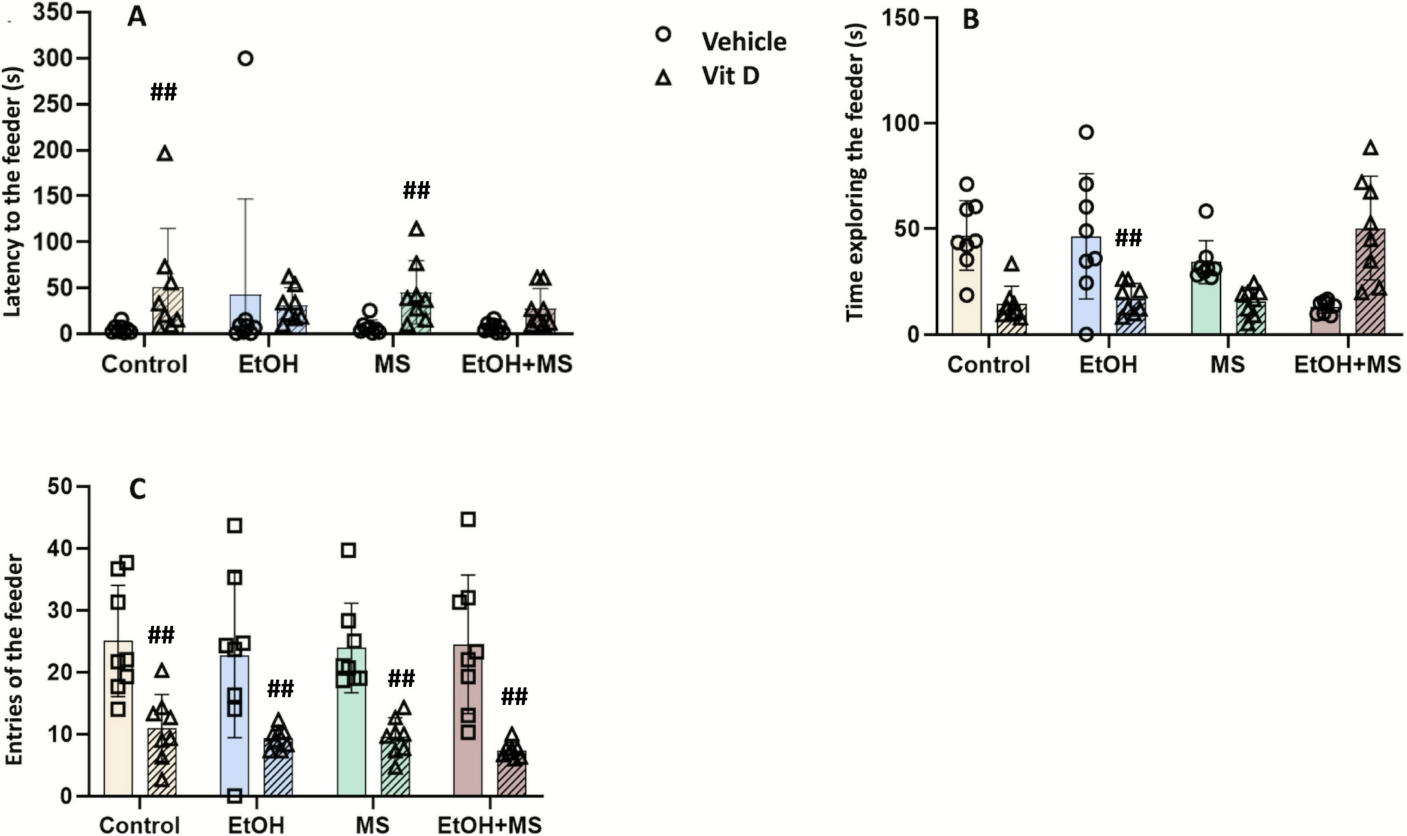
Reward omission–induced responding following removal of expected reward. Latency to explore the feeder (A). Time spent exploring the feeder (B). Number of entries into the feeder (C). Values are expressed as mean ± SEM (8 animals/group). ##*p* < 0.01 compared to the corresponding vehicle‐treated group in the same experimental condition, as indicated. Abbreviations: EtOH, ethanol; EtOH + MS, ethanol plus maternal separation; MS, maternal separation; VitD, vitamin D.

To evaluate the impact of vitamin D on emotional reactivity following the omission of an expected reward, rats were tested in a reward omission paradigm in which the feeder previously associated with palatable food now contained only nonedible plastic beads (Figure 3). This setup preserved the spatial and contextual cues from the prior motivational test but removed reinforcement, allowing assessment of persistent feeder‐directed responding under nonreinforcement.

Multifactorial ANOVA revealed a significant main effect of treatment on latency to initiate feeder exploration [*F*(1.55) = 21.24, *p* < 0.001] (Figure 3A). Post hoc analysis indicated that rats treated with vitamin D alone (saline + nMS + VitD) exhibited a longer latency to approach the empty feeder compared to controls (saline + nMS + vehicle) (*p* < 0.01), suggesting reduced persistence in feeder‐directed responding when reinforcement was absent. Similarly, the MS + VitD group displayed significantly longer latencies relative to its vehicle‐treated counterpart (*p* < 0.01), consistent with decreased perseverative responding under reward omission.

A significant main effect of treatment was also observed for the total time spent exploring the feeder [*F*(1.55) = 71.15, *p* < 0.001] (Figure 3B). Post hoc analysis revealed that vitamin D supplementation significantly reduced exploration time in ethanol‐exposed animals compared to their vehicle‐treated counterparts (*p* < 0.01). No significant differences were detected between vehicle‐treated groups or between other vitamin D‐treated conditions, indicating that the effect of vitamin D on exploration time was specific to ethanol exposure. Regarding the number of feeder entries (Figure 3C), a significant main effect of treatment was observed [*F*(1.55) = 75.77, *p* < 0.001]. The highest number of entries occurred in the control group, consistent with baseline exploratory behaviour. All vitamin D‐treated groups exhibited fewer entries compared to their respective vehicle controls (*p* < 0.01), indicating reduced repetitive feeder‐directed responding under nonreinforcement conditions.

### 3.3 Effect of Vitamin D on Hippocampal Neuronal Differentiation

**FIGURE 4:**
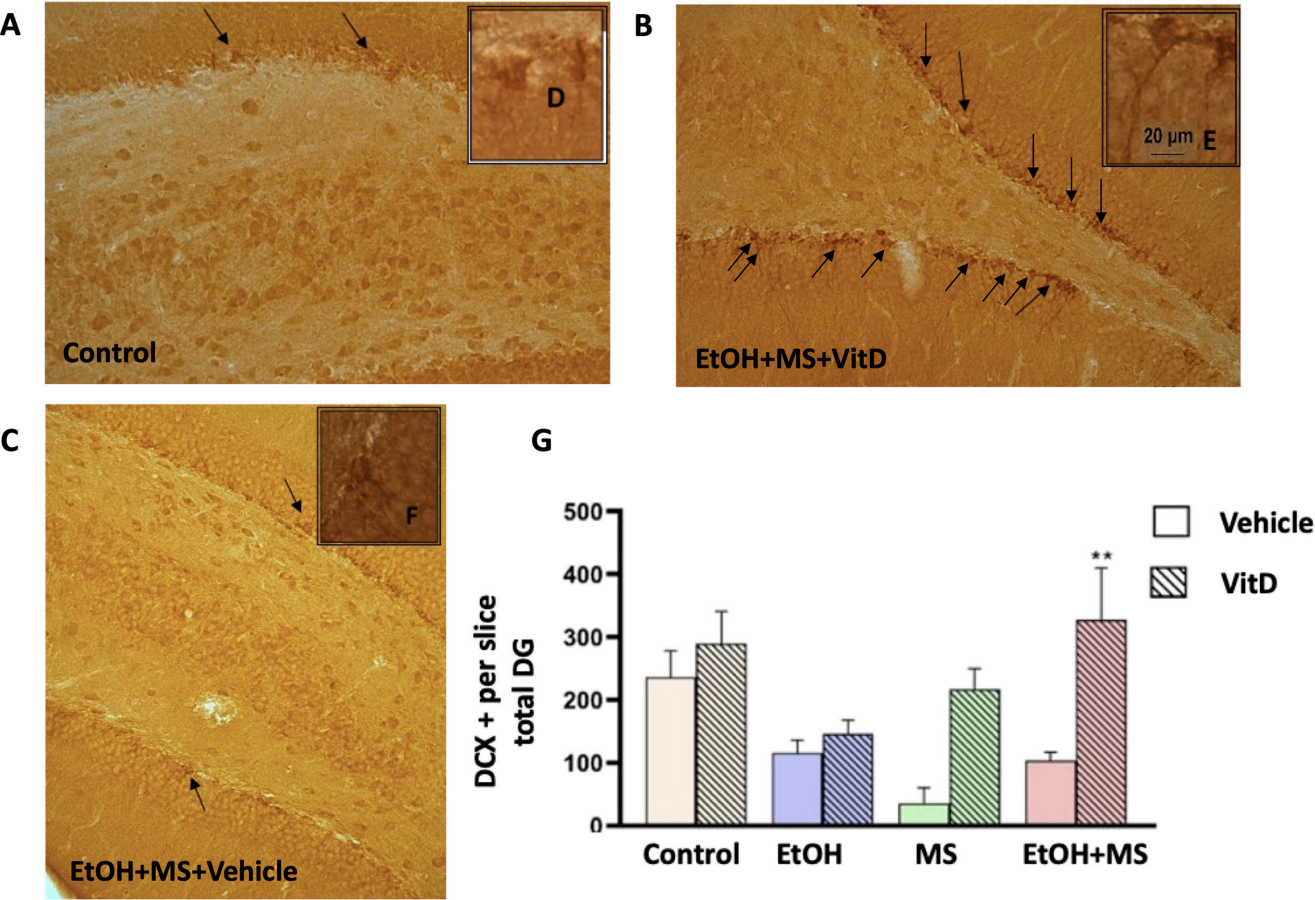
Neuronal differentiation in the DG across experimental groups. (A–C) Representative photomicrographs of doublecortin (DCX)‐positive cells in the DG: (A) control group; (B) EtOH + MS + VitD; (C) EtOH + MS + vehicle. Scale bar = 100 μm*.* (D–F) High‐magnification images showing the typical morphology of DCX‐positive neuroblasts: (D) control; (E) EtOH + MS + VitD; (F) EtOH + MS + Vehicle. Scale bar = 20 μm*.* (G) Quantification of the number of DCX‐positive cells in the DG. Data are expressed as mean ± SEM (*n* = 5–6 animals per experimental group; values represent the mean number of DCX+ cells per section (1:6 series) averaged within each animal). ##*p* < 0.01 compared to the corresponding group without vitamin D treatment. Abbreviations: DCX, doublecortin; DG, dentate gyrus; EtOH, ethanol; EtOH + MS, ethanol plus maternal separation; MS, maternal separation; VitD, vitamin D.

To investigate whether vitamin D modulates neuronal differentiation following early exposure to alcohol and stress, immunohistochemical labelling of doublecortin (DCX)—a marker of immature neurons—was performed in the DG of the hippocampus. Quantification of DCX‐positive cells was conducted along the subgranular zone (SGZ), the neurogenic niche of the DG (Figure 4). Multifactorial ANOVA revealed a significant main effect of treatment [*F*(1.35) = 8.27, *p* < 0.01], indicating that vitamin D influenced hippocampal neurogenesis. Additionally, there was a significant interaction between condition (EtOH vs. saline) and stress (MS vs. nMS) [*F*(1.35) = 6.51, *p* < 0.05], suggesting that the combined impact of ethanol exposure and early‐life stress modulated the neurogenic response. Post hoc analyses showed that the EtOH + MS group treated with vitamin D exhibited a significant increase in the number of DCX + cells compared to its corresponding nontreated group (*p* < 0.01). This finding indicates that vitamin D supplementation during adolescence attenuates the reduction in hippocampal neurogenesis induced by the dual exposure to ethanol and early‐life stress.

### 3.4 Effect of Vitamin D on Dendritic Arborization of Granule Cells in the Dentate Gyrus

**FIGURE 5:**
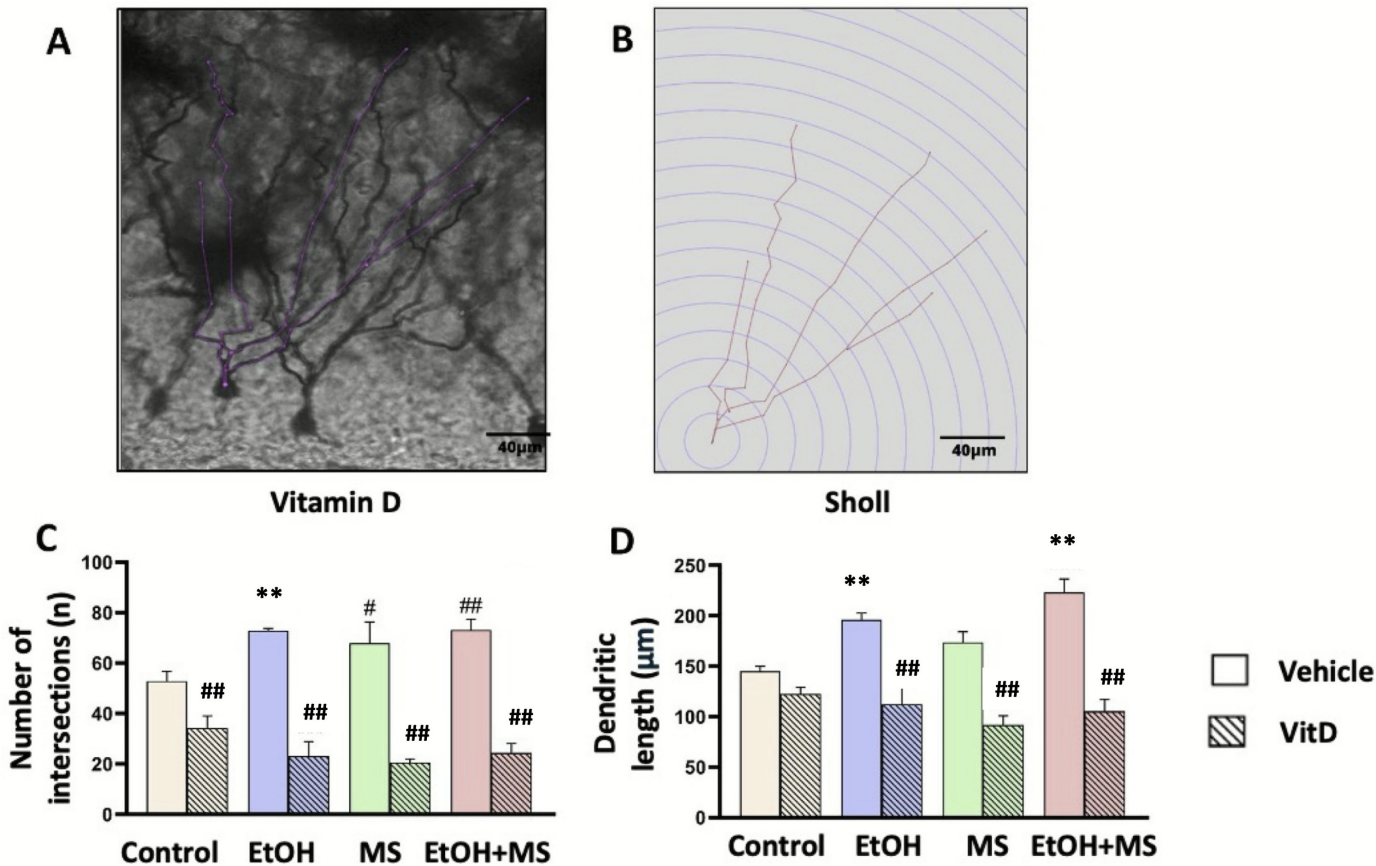
Dendritic arborization in the dentate gyrus across experimental groups. (A) Representative photomicrograph of a granule cell in the DG stained with the Golgi‐Cox method in a vitamin D‐treated animal. (B) Sholl analysis illustrating dendritic branching distribution. (C) Quantification of the total dendritic intersections. (D) Maximum dendritic length. Data are expressed as mean ± SEM (*n* = 3 animals per group; values represent animal‐level means derived from 5 neurons per animal). ##*p* < 0.01 compared to corresponding group without vitamin D; *p < 0.05, **p < 0.01 compared to control group. Abbreviations: EtOH, ethanol; EtOH + MS, ethanol plus maternal separation; MS, maternal separation; VitD, vitamin D.

Sholl analysis was performed on Golgi‐Cox–impregnated granule cells from the DG (Figure 5A) to assess the effects of vitamin D supplementation on dendritic arborization (Figure 5B). All dendritic analyses were conducted using animal‐level means derived from Golgi‐impregnated neurons (*n* = 3 animals per experimental group). For the total number of dendritic intersections (Figure 5C), multifactorial ANOVA revealed a main effect of treatment [*F*(1.16) = 152.65, *p* < 0.001], indicating an association between vitamin D treatment and differences in dendritic complexity. Significant interactions were also observed between ethanol exposure and treatment [*F*(1.16) = 5.99, *p* = 0.026] and between stress and treatment [*F*(1.16) = 4.93, *p* = 0.041], suggesting that the effects of vitamin D on dendritic branching were modulated by both early‐life ethanol exposure and maternal separation. Post hoc analyses indicated that animals exposed to EtOH, MS or both (EtOH + MS) exhibited an increased number of dendritic intersections compared to controls (*p* < 0.01), suggesting a pattern of maladaptive dendritic overgrowth. Notably, vitamin D supplementation significantly reduced the number of intersections in all experimental groups compared to their respective vehicle‐treated counterparts (*p* < 0.001), indicating attenuation of this maladaptive dendritic remodelling. For maximum dendritic length (Figure 5D), ANOVA revealed a significant main effect of ethanol exposure [*F*(1.16) = 10.05, *p* = 0.006], indicating that EtOH independently affected dendritic elongation. Furthermore, significant interactions were detected between ethanol and treatment [*F*(1.16) = 8.71, *p* = 0.009] and between stress and treatment [*F*(1.16) = 8.12, *p* = 0.011], reinforcing the modulatory influence of vitamin D in the context of early‐life insults.

Taken together, these findings indicate that vitamin D supplementation during adolescence modulates dendritic structure in the DG, reversing the excessive dendritic branching and elongation induced by early‐life ethanol exposure and maternal separation.

## 4 Discussion

Early‐life experiences profoundly shape neural, behavioural, and psychological development, with enduring consequences across the lifespan. During critical developmental windows such as infancy and childhood, heightened neural plasticity is marked by intense synaptogenesis, dendritic remodelling and circuit refinement (Smith and Pollak 2020). In this study, we modelled two early‐life stressors commonly observed in clinical contexts: early postnatal ethanol exposure, simulating third trimester‐equivalent brain development and maternal separation, a validated model of psychosocial stress. We aimed to assess whether vitamin D supplementation during adolescence—a period of significant synaptic remodelling and maturation of limbic‐prefrontal circuits (Spear 2000; Andersen 2003)—could mitigate behavioural and neuroplastic impairments induced by these early insults.

In the palatable food intake test, early ethanol exposure increased latency to initiate eating, consistent with reduced hedonic drive. This finding aligns with reports of disrupted reward circuitry following developmental alcohol exposure (Marquardt et al. 2020). Vitamin D supplementation reduced this latency, suggesting an improvement in task performance, consistent with evidence linking vitamin D to modulation of dopaminergic pathways and reward‐related processing (Turner et al. 2013; Peak et al. 2015).

Vitamin D also enhanced both the time spent eating and the amount of palatable food consumed, particularly in the EtOH + MS group, indicating increased engagement with the rewarding stimulus. While these findings reflect restored motivation, future studies using effort‐based paradigms may better distinguish adaptive motivation from habitual or passive consumption.

In the reward omission task, vitamin D modulated feeder‐directed responding following the omission of an expected reward in a context‐dependent manner, evidenced by increased latency and fewer entries into the feeder across conditions and by reduced feeder exploration time in animals exposed to ethanol alone. These behavioural changes are indicative of altered responding under nonreinforcement conditions, which may reflect changes in behavioural adaptation or extinction‐related processes rather than a global effect on emotional reactivity, without inferring a specific emotional state. This interpretation is consistent with classical frustration theory (Amsel and Roussel 1952) and suggests involvement of hippocampal–prefrontal circuits in a manner dependent on the nature of early‐life insults.

Histological analyses revealed that EtOH + MS reduced the number of DCX‐positive neuroblasts in the DG, indicating impaired hippocampal neurogenesis. Vitamin D supplementation was associated with increased DCX‐positive cell counts in this group, in line with previous reports describing neurogenic effects of vitamin D signalling (Shirazi et al. 2015; Morello et al. 2018). Interestingly, both EtOH and MS exposure increased dendritic complexity and length in granule cells. Although dendritic growth is often linked to adaptive plasticity, increases in dendritic arborization following early‐life stress have been interpreted as potentially maladaptive in some contexts, reflecting premature differentiation or stabilization of neural circuits (Bath et al. 2016). These alterations have been linked to reduced cognitive flexibility and impaired emotional regulation. Supporting this, early‐life stress is known to downregulate Wnt signalling and promote early maturation of hippocampal neurons (Sambo et al. 2022). Vitamin D treatment was associated with attenuation of these dendritic alterations, suggesting a potential alignment toward typical developmental patterns. These effects may involve modulation of neurotrophic signalling pathways, including BDNF and Wnt/*β*‐catenin, as well as anti‐inflammatory mechanisms (Eyles et al. 2013; Groves et al. 2014).

The convergence of behavioural and structural findings points to hippocampal plasticity as a potential substrate underlying the observed behavioural effects. The DG plays a central role in motivational behaviour, stress regulation and cognitive processing (Anacker and Hen 2017; Snyder et al. 2011). Alterations in neurogenesis and dendritic organization within this region may contribute to changes in behavioural performance, particularly under conditions of early‐life adversity. These findings underscore adolescence as a sensitive developmental window during which interventions may influence neuroplastic trajectories. Given the protracted maturation of hippocampal and prefrontal circuits during this period, adolescent interventions may offer opportunities to modify outcomes associated with early‐life insults.

Nonetheless, some limitations should be acknowledged. This study was not powered to detect sex‐specific effects, and sex was therefore not included as a variable in the statistical analyses. The alcohol exposure model was restricted to third trimester–equivalent timing, limiting extrapolation to earlier prenatal exposures. Additionally, while behavioural assays focused on motivation and reward omission–induced responding, other cognitive and affective domains were not assessed. Repeated handling and daily intraperitoneal injections during adolescence may also have influenced stress responsivity, behaviour or neuroplastic outcomes and should be considered when interpreting the findings. Future studies could consider alternative routes of vitamin D administration, such as oral supplementation to minimize potential effects related to repeated handling and injections. Because ethanol exposure overlapped with the maternal separation window, acute ethanol effects may have modulated pup‐level stress responsivity during separations; future studies incorporating physiological and behavioural stress‐related measures (e.g., corticosterone levels, pup distress vocalizations and/or maternal behaviour) would help to disentangle these influences. It should also be noted that DCX immunoreactivity reflects the presence of immature neurons and does not allow dissociation between changes in cell proliferation, survival or maturation. Future studies employing birthdating approaches, such as BrdU or EdU labelling, will be necessary to further dissect the cellular mechanisms underlying the observed effects. Finally, the maternal separation protocol employed represents a specific model of early‐life stress and may not capture the full spectrum of adverse developmental experiences; alternative stress paradigms and developmental windows should be explored in future studies to assess the generalizability of the observed findings.

## Conclusion

In summary, our results demonstrate that vitamin D supplementation during adolescence is associated with improvements in behavioural performance and neuroplasticity measures in animals exposed to early‐life ethanol and maternal separation. Vitamin D was associated with reduced anhedonia‐like responding, context‐dependent modulation of reward omission‐induced responding, increased hippocampal neurogenesis and attenuation of alterations in dendritic architecture. Together, these findings suggest that vitamin D supplementation during adolescence may modulate neurobehavioural and structural outcomes following early‐life insults, supporting its potential relevance as an adjunctive strategy for neurodevelopmental conditions associated with foetal alcohol exposure and early‐life stress. Future studies should aim to dissect the underlying molecular mechanisms and evaluate the translational relevance of these findings in clinical populations.

## Funding

This work was supported by the Fundação de Amparo à Pesquisa e Inovação do Estado de Santa Catarina (FAPESC) under the Universal Research Program—Public Call No. 12/2020, Grant Agreement No. 2021TR1523.

## Conflicts of Interest

The authors declare no conflicts of interest.

## Data Availability

The datasets generated and analysed during the current study are available from the corresponding author upon reasonable request.
